# The Impact of Adolescent Body Weight Misclassification on Dieting Behaviour From Adolescence to Adulthood: A Longitudinal Study

**DOI:** 10.1002/hpja.70185

**Published:** 2026-04-14

**Authors:** Abdulaziz D. Aloufi, Jake M. Najman, Abdullah A. Mamun

**Affiliations:** ^1^ School of Public Health The University of Queensland Brisbane Australia; ^2^ Madinah Health Cluster, Ministry of Health Madinah Saudi Arabia; ^3^ School of Social Science The University of Queensland Brisbane Australia; ^4^ Poche Centre for Indigenous Health The University of Queensland Brisbane Australia

**Keywords:** adolescent, adult, body weight, diet, weight perception

## Abstract

**Introduction:**

Weight misclassifications and consequent uptake of weight loss activities may influence future eating habits and weight trajectories. This study examines the longitudinal association between body weight misclassifications in adolescence and its impact on dieting patterns from adolescence to adulthood.

**Methods:**

Data were retrieved from an Australian longitudinal birth‐cohort study. The analytical sample (*n* = 1132) comprises participants whose measured and perceived body weight at 14 years of age were recorded and prospectively provided dieting information at 14, 21 and 30 years of age. Weight misclassification was determined by comparing measured body mass index with perceived body weight.

**Results:**

The proportion of participants who reported dieting increased from 19% at 14‐year follow‐up to 48% at 30‐year follow‐up. Among adolescents who reported dieting, 72% reported dieting again at 21‐year follow‐up and among those who reported dieting at the 21‐year follow‐up, 81% reported dieting at 30‐year follow‐up. Adolescents who overestimated their body weight had threefold higher odds of dieting compared to their counterparts at that age and were more likely to follow persistent dieting patterns into adulthood. After stratifying by BMI categories, normal weight adolescents who overestimated their weight had higher odds of dieting compared with normal weight adolescents who correctly estimated their weight; however, there was no significant association between weight underestimation and dieting behaviour.

**Conclusions:**

The prevalence of dieting behaviour increased from adolescence to adulthood. Adolescent weight overestimation was the most significant factor associated with dieting over the study period.

**So What?:**

Interventions targeting weight misclassification during adolescence may help prevent the adoption of unhealthy dieting behaviours.

**Abbreviations**
adjust: adjusted
BMI: body mass index
CI: confidence interval
F/U: follow up
MUSP: Mater‐University of Queensland Study of Pregnancy and its outcomes
OR: odds ratio
Unadj.: unadjusted
*χ* ^2^
: chi‐square test

## 1 Introduction

Adolescence is a life stage where social desirability and peer acceptability, particularly regarding body shape and weight play a key role in emotional and mental health [1, 2]. During this stage, individuals may hold weight related perceptions that influence dietary practices and weight management behaviours across the life course [3, 4, 5, 6]. Self‐perception of overweight or obesity, irrespective of actual body mass index (BMI), is a key influencer for pursuing weight loss activities [7, 8, 9]. Studies exploring the association between adolescent body weight misclassification and dieting behaviours [5, 8, 10, 11] report that correct body weight estimation contributes positively to behaviour promoting weight control, for example, restrained eating [3, 9, 12]. Thus, studying body weight misclassification during adolescence is essential for identifying early risk factors for unhealthy dieting and weight control behaviours.

In contrast, underestimation of body weight can be a deterrent to weight loss because individuals might downplay their weight gain [3, 4, 13, 14]. Several studies conducted among adolescents with overweight and obesity indicate that weight underestimation is relatively common in this group and may reduce engagement in recommended weight management behaviours [3, 6, 9, 15]. Similarly, a large cross‐sectional study of adults with overweight and obesity found that body weight underestimation is associated with less intent to lose weight and less physical activity compared to correct body weight estimation [14]. This was further supported in a longitudinal study reporting that even after adjusting for key covariates, individuals who correctly identify themselves as overweight or obese are less likely to experience weight gain than those who incorrectly perceive themselves as normal weight [16].

Some studies have reported that adolescents who overestimate their body weight are more likely to adopt weight control behaviours, although these behaviours may not necessarily be health‐promoting [7, 9]. Many studies highlight that body weight overestimation is associated with more extreme weight control practices including fasting for more than 24 h and the use of diet pills [5, 17, 18]. In a 9‐year follow‐up study of school‐aged children across 24 countries, it was reported that perceiving oneself as overweight is associated with promoting dieting behaviours [19]. The use of extreme measures for weight loss, particularly in those individuals with a healthy weight, is linked with poor physical health, immunosuppression and increased risk of cardiovascular condition [20]. This evidence points to the importance of correct estimation of body weight and the use of appropriate activities for weight management.

Considering that adolescence, a critical life stage, significantly shapes the development of healthy habits and behaviours over the full life span, weight misclassification and consequent uptake of weight loss activities, such as dieting, can play a significant role in influencing future food habits [12, 21]. Therefore, assessing the prevalence of weight misclassification in adolescence and its role in long‐term dieting behaviours can help in establishing the long‐term impact of weight misclassification in adolescence. Studying the longitudinal implications of weight misclassification among adolescents on dieting behaviour is important because inaccurate weight perception is potentially modifiable. Early identification of weight misclassification during adolescence may inform health promotion strategies aimed at improving accurate body weight perception and ultimately reducing engagement in unhealthy dieting practices. Unfortunately, none of the previous studies on body weight misclassification explored the long‐term effect of body weight misclassification, particularly in the context of dieting. Addressing the gaps in the literature, this paper is based on a birth cohort study to explore the longitudinal association between body weight misclassification during the adolescent period with dieting behaviour from this stage to adulthood.

## 2 Method

The data from the participants of the Mater‐University of Queensland Study of Pregnancy (MUSP) were used for this study. MUSP is a prospective cohort study of pregnant women who received antenatal care at Mater Misericordiae Hospital in Brisbane, Australia, between 1981 and 1984. The mothers and their offspring have been followed up prospectively at the birth of the study child, 3–5 days post‐delivery, 6 months (1981–1984), 5 years (1986–1988), 14 years (1995–1997), 21 years (2001–2004), 27 years for mothers (2008–2011) and 30 years for offspring (2010–2014). At the 21‐year and 30‐year follow‐ups, offspring completed details about themselves. At 14‐year follow‐up visit, both mothers and children completed children's questionnaires. These details include factors that influence mothers and their children's dieting related behaviours including sociodemographic, psychological, lifestyle and physical developmental factors. More details about this study have been reported elsewhere [22, 23].

In this study, the main analytical sample comprises of 1132 subjects for whom measured and perceived body weight data at the 14‐year follow‐up as well as dieting information at 14‐year, 21‐year and 30‐year follow‐ups were available. Up to the 14‐year follow‐up, written informed consent was obtained from mothers for their participation and for their children's information while offspring gave their own informed consent at the 21‐year and 30‐year follow‐ups.

### 2.1 Measurements

#### 2.1.1 Dieting Variables

**FIGURE 1:**
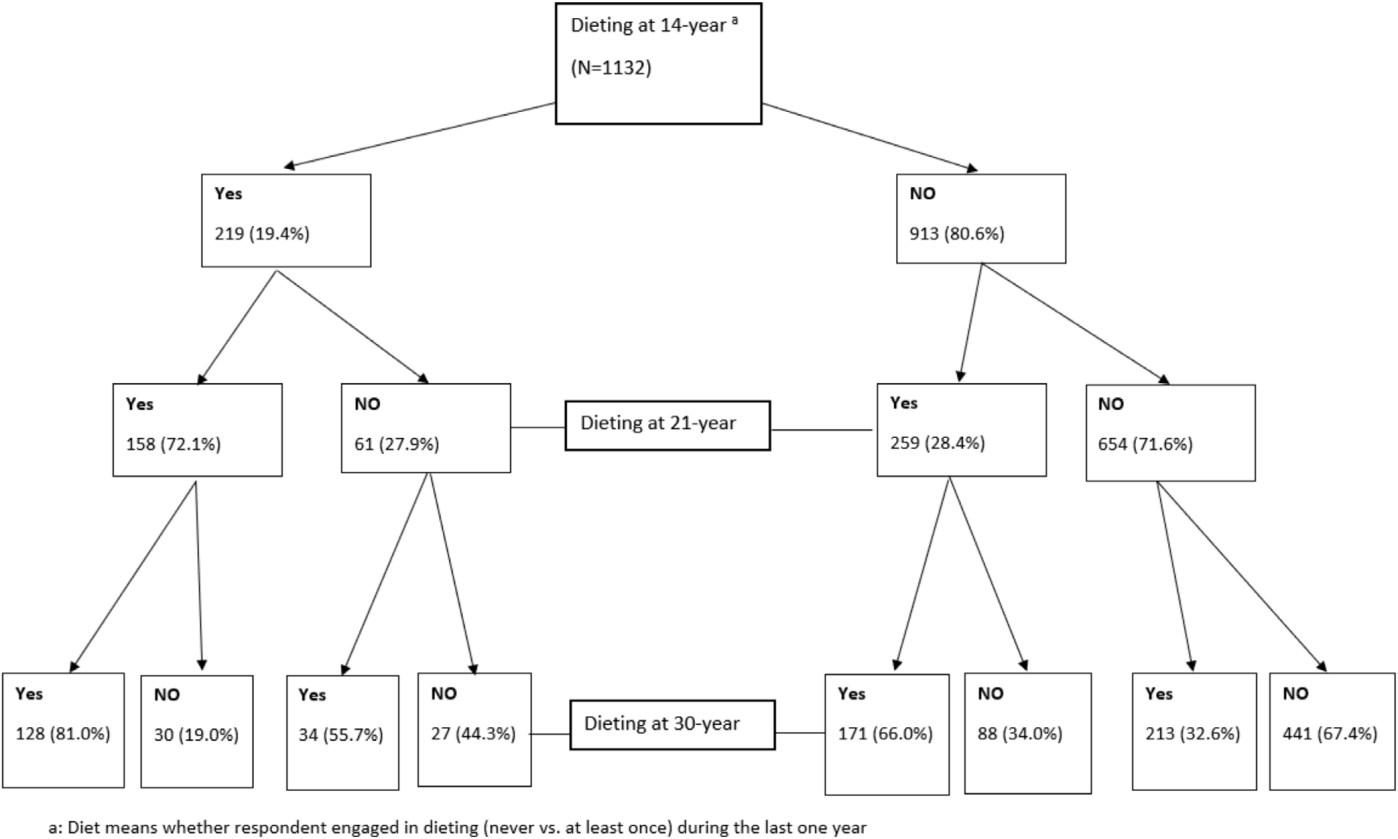
Longitudinal tracking of participants (*n*, %) who were dieting to lose body weight at 14‐year, 21‐year and 30‐year follow‐ups (*N* = 1132) using data from the Mater‐University of Queensland Study of Pregnancy.

Dieting information was retrieved from participants' questionnaires at 14‐year, 21‐year and 30‐year follow‐ups. Similar questions were asked at each follow‐up. At the 14‐year follow‐up, adolescent dieting behaviour was assessed with the question ‘How often do you go on a diet to lose weight?’ The response categories for this question were ‘most of the time’, ‘a few times a year’, ‘rarely’ or ‘never.’ For the purpose of analysis, participants who reported they dieted ‘most of the time’ and ‘a few times a year’ were grouped together, whereas those who reported they dieted ‘rarely’ and ‘never’ were merged into a single category. At the 21‐year and 30‐year follow‐ups, the same questions were asked at each follow‐up, which is ‘How often have you gone on a diet to lose weight during the last year?’ With response options ‘never’, ‘1–4 times’, ‘5–10 times’, ‘more than 10 times’ or ‘I am always on a diet to lose weight’. For the purpose of analysis, the latter four options (1–4 times, 5–10 times, more than 10 times and always on dieting) were merged into a single category and was labelled as ‘dieting’. After summing dieting and non‐dieting cases at all follow‐ups, we created a new variable which contains eight categories (see Figure 1):

(1) ‘dieting at each study phase’, (2) ‘never diet at any phase’, (3) ‘dieting only at 14‐year follow‐up’, (4) ‘dieting only at 21‐year follow‐up’, (5) ‘dieting only at 30‐year follow‐up’, (6) ‘dieting only at 14‐year and 21‐year follow‐ups’, (7) ‘dieting only at 14‐year and 30‐year follow‐ups’, (8) ‘dieting only at 21‐year and 30‐year follow‐ups’.

The wording of the dieting questions differed between the 14‐year follow‐up and the 21‐year and 30‐year follow‐ups to ensure age‐appropriate phrasing and to standardise the recall period in adulthood. Across all follow‐ups, the questions were designed to capture intentional dieting for the purpose of weight loss [24, 25].

#### 2.1.2 Body Weight Misclassification at 14‐Year Follow‐Up

##### 2.1.2.1 Measured Body Weight at the 14‐Year Follow‐Up

BMI was calculated from measured weight and height. Weight was measured using a scale accurate to 0.2 kg with participants lightly clothed. The average of two measures was recorded. Height was measured using a portable stadiometer. Overweight and obesity cut off values were identified according to Cole et al. international definition for overweight and obesity [26]. For the main analysis, overweight and obese categories were grouped into a single category called ‘overweight and obese’ due to small numbers in the obese group. Subjects who fell under 10th BMI percentile were recorded as ‘underweight’, others are categorised as ‘normal weight’.

Weight perception at the 14‐year follow‐up was assessed by asking the adolescents ‘Do you think of yourself as’ The response categories for this question were ‘very underweight’, ‘slightly underweight’, ‘about the right weight’, ‘overweight’ or ‘very overweight’. The first two and last two categories were merged into ‘underweight’ and ‘overweight and obese’ categories, respectively, to make them compatible with the three BMI categories.

##### 2.1.2.2 Weight Misclassification

Measured body weight categories were compared with weight perception categories to identify body weight misclassification. This approach, based on comparing BMI‐derived weight status with self‐perceived weight, has been widely used to define body weight misclassification in weight perception research [6, 27, 28]. Weight misclassification comprises three categories ‘correct estimation’, ‘underestimation’, and ‘overestimation’. Correct estimation is when the body weight perception category is concordant with the measured body weight category (e.g., respondents with overweight and obesity who recorded their perceived weight as overweight). Underestimation is when the body weight perception category is reported to be lower than the measured body weight category (e.g., respondents with overweight and obesity who recorded their perceived weight as normal or underweight). Overestimation occurs when the body weight perception category is reported to be higher than the measured body weight category (e.g., normal weight respondents who recorded their perceived weight as overweight and obese).

### 2.2 Covariates

The following factors were included in the data analysis based on evidence that they were associated with going on a diet to lose weight or body weight misclassification [8, 9, 24, 29, 30]. These factors were considered as covariates and are grouped according to the data collection period (Table S1).

Maternal weight and height in pregnancy, maternal education and parental racial origin data were reported at baseline data collection. Maternal BMI cut off values were defined according to the WHO definition for BMI [31]; maternal education was coded into ‘incomplete high school’, ‘completed high school’ or ‘post high school’; and parental racial origin was divided into ‘White’, ‘Asian’, or ‘Aboriginal‐Islander’. Child sex was recorded as ‘male’ or ‘female’ at the birth follow‐up.

At the 14‐year follow‐up, adolescents' participation in organised sports during the last week was categorised into ‘0–1 day’, ‘2–3 days’, ‘4–5 days’, or ‘6–7 days’. Pubertal development stages were divided into five stages based on the Tanner scale [32, 33]. Family eating together was categorised into ‘at least once a day’; ‘a few times a week’; ‘about once a week’ or ‘less than once a week’.

### 2.3 Statistical Analyses

Longitudinal frequency distribution of dieting information at 14‐year, 21‐year and 30‐year follow‐ups are described in a flowchart (Figure 1). To determine the frequency of weight misclassification, body weight perception categories were matched with BMI‐measured body weight categories at the 14‐year follow‐up.

At each follow‐up, we used bivariate analyses and the chi‐square test (*χ* ^2^) to estimate the association between dieting to lose weight with each of the following factors at 14 years: BMI, body weight perception and body weight misclassification. In logistic regression analysis [34], the association between weight misclassification and dieting was estimated using of odds ratio (OR) and 95% confidence intervals at each follow‐up. Then, the latter analysis has been repeated after we included the previously mentioned potential confounders. The covariates included in the adjusted logistic regression models were selected a priori based on prior evidence (Table S1) and were not determined by the results of bivariate analyses.

For the longitudinal analysis, multinomial logistic regression was used to estimate OR and 95% confidence intervals between weight misclassification at the 14‐year follow‐up with the dieting pattern (eight categories described in Figure 1, no dieting at any follow‐up as the reference group) from 14‐year to 30‐year follow‐up. Only five categories of dieting variable were included in the analyses (‘dieting at each phase’, ‘never diet at any phase’, ‘dieting only at 21 years and 30 years’, ‘dieting only at 21 years’ and ‘dieting only at 30 years’) with ‘never diet at any phase’ being the base category. The other three categories were excluded due to small numbers.

Additionally, stratified analyses were conducted to examine the associations between weight misclassification and dieting separately for adolescents classified as normal weight and those classified as overweight and obese (Tables S2 and S3). Stratified models for adolescents in the underweight group were not presented due to very small numbers. Furthermore, baseline BMI was not included as a covariate because BMI status determines the possible direction of misclassification, which would have resulted in sparse data and unstable parameters if included in the models.

All data analyses were undertaken using SAS version 9.4 (SAS Institute Inc., Cary, NC, USA) [35].

## 3 Results

Figure 1 shows the longitudinal tracking of dieting from 14‐ to 21‐ year and then to 30‐year follow‐up. Of the 1132 participants, the proportion of those who reported dieting to lose weight increased from 19.4% at the 14‐year follow‐up to 36.8% at the 21‐year follow‐up and 48.2% at the 30‐year follow‐up. Among participants who reported dieting at 14 years of age, around 72% of them reported dieting again at the 21‐year follow‐up; furthermore, approximately 81% of those who were dieting at 21 years reported dieting at the 30‐year follow‐up. Regarding the participants who reported dieting at 21 years of age but were not dieting at 14 years, two thirds of them reported dieting at the 30‐year follow‐up as well. Almost a third of respondents who had never dieted at the 14‐year and 21‐year follow‐ups, began dieting at the 30‐year follow‐up. Of those who were not dieting at the 14‐year follow‐up, 72% did not report dieting at the 21‐year follow‐up. The trend for continuity of not dieting persisted at the 30‐year follow‐up, where 67% of the participants reported not dieting.

Table 1 shows the cross‐sectional association between BMI, weight perception and weight misclassification at 14 years and dieting at each follow‐up. There are consistent associations between weight misclassification at the 14‐year follow‐up and dieting. Dieting prevalence increased as BMI level increased at each follow‐up. Similar trends appeared in relation to dieting and weight perception. At each follow‐up, underweight or self‐reported underweight participants were the least likely to report dieting, while those who were overweight and obese or self‐reported overweight and obesity were the most likely to report dieting. Also, dieting behaviour varied among weight misclassification groups. At each follow‐up, dieting behaviour was reported more often among the overestimation group. For example, almost a third of respondents who overestimated their body weight were on a diet while only 13.1% among the correct estimation group reported dieting at the 14‐year follow‐up.

**TABLE 1:** Association of body mass index (BMI), perceived body weight and weight misclassifications at 14 years of age with dieting to lose weight at 14, 21 and 30 years of age (*N* = 1132) using data from the Mater‐University of Queensland Study of Pregnancy.

BMI at 14 year	Total (*n*)	Dieting at 14 years F/U^a^, (%), *n*			Dieting at 21 years F/U^b^, (%), *n*			Dieting at 30 years F/U^b^, (%) *n*		*p* *
		Not dieting	Dieting	*p* *	Not dieting	Dieting	*p* *	Not dieting	Dieting	
Underweight	115	(98.3%) 113	(1.7%) 2	< 0.0001	(89.6%) 103	(10.4%) 12	< 0.0001	(69.6%) 80	(30.4%) 35	< 0.0001
Normal weight	773	(85.8%) 664	(14.2%) 110		(67.0%) 518	(34.0%) 255		(55.6%) 430	(44.4%) 343	
Overweight	244	(56.2%) 137	(43.9%) 107		(38.5%) 94	(61.5%) 150		(31.1%) 76	(68.9%) 168	
Weight perception at 14 year										
Underweight	158	(94.9%) 150	(5.1%) 8	< 0.0001	(84.8%) 134	(15.2%) 24	< 0.0001	(67.1%) 106	(32.9%) 52	< 0.0001
Normal weight	580	(93.1%) 540	(6.9%) 40		(71.9%) 417	(28.1%) 163		(60.0%) 348	(40.0%) 232	
Overweight	394	(56.6%) 223	(43.4%) 171		(41.6%) 164	(58.4%) 230		(33.5%) 132	(66.5%) 262	
Weight misclassification at 14 year										
Correct estimation	664	(86.9)577	(13.1%) 87	< 0.0001	(66.4%) 441	(33.6%) 223	0.01	(54.4%) 361	(45.6%) 303	0.07
Underestimation	217	(76.5%) 166	(23.5%) 51		(61.3%) 133	(38.7%) 84		(50.7%) 110	(49.3%) 107	
Overestimation	251	(67.7%) 170	(32.3%) 81		(56.2%) 141	(43.8%) 110		(45.8%) 115	(54.2%) 136	

^a^ At the 14‐year follow‐up, adolescent dieting behaviour was assessed with the question ‘How often do you go on a diet to lose weight?’ with response options ‘most of the time’, ‘a few times a year’, ‘rarely’ or ‘never’. For the purpose of analysis, participants who reported they dieted ‘most of the time’ and ‘a few times a year’ were grouped together as dieting at 14‐year follow‐up, whereas those who reported they dieted ‘rarely’ and ‘never’ were grouped together as not dieting at 14‐year follow‐up.

^b^ At the 21‐year and 30‐year follow‐ups, the same questions were asked at each follow‐up which is ‘how often have you gone on a diet to lose weight during the last year?’ with response options ‘never’, ‘1–4 times’, ‘5–10 times’, ‘more than 10 times’ or ‘I am always on a diet to lose weight’. For the purpose of analysis, we merged the latter four options (1–4 times, 5–10 times, more than 10 times and always on a diet) into a single category as dieting and never as not dieting.

* *p* value indicates the significance level of the difference between two categorical variables. We used a Chi‐squared test for categorical data.

Table 2 shows the odds ratio (OR) and 95% confidence interval (95% CI) estimation between weight misclassification and dieting at each follow‐up. At the 14‐year follow‐up, adolescents who underestimated their body weight had two times higher odds of dieting (OR: 2.04; 95% CI: 1.39, 3.0) compared to those who correctly estimated their body weight. Adolescents who overestimated their body weight had three times higher odds (OR: 3.16; 95% CI: 2.23, 4.47) of engaging in dieting compared to their correct estimation counterparts. These results remained significant for underestimation and overestimation of body weight in the adjusted models at the 14‐year follow‐up.

**TABLE 2:** The association between weight misclassification at 14‐year follow‐up and dieting to lose weight at 14‐year, 21‐year and 30‐year follow‐ups (*N* = 1132) using data from the Mater‐University of Queensland Study of Pregnancy.

Predictor	*N*	Not dieting	Dieting at 14 years F/U		Dieting at 21 years F/U		Dieting at 30 years F/U	
			Dieting		Dieting		Dieting	
			Unadj. OR (95% CI)	Adjust. OR^a^ (95% CI)	Unadj. OR (95% CI)	Adjust. OR^a^ (95% CI)	Unadj. OR (95% CI)	Adjust. OR^a^ (95% CI)
Misclassification at 14 years F/U								
Correct estimation (reference)	664	1.00	1.00	1.00	1.00	1.00	1.00	1.00
Underestimation	217	1.00	2.04 (1.39, 3.0)*	2.15 (1.39, 3.33)*	1.25 (0.91, 1.72)	1.22 (0.83, 1.79)	1.16 (0.85, 1.58)	1.09 (0.77, 1.53)
Overestimation	251	1.00	3.16 (2.23, 4.47)*	2.86 (1.98, 4.14)*	1.54 (1.15, 2.08)*	1.12 (0.80, 1.56)	1.41 (1.05, 1.89)*	1.25 (0.91, 1.70)

^a^ Adjusted OR: Sex, race, maternal education, maternal BMI at baseline follow‐up; exercise, family eating together and pubertal development at 14 years of age.

* Results are statistically significant with *p* value < 0.05.

Table 3 shows the longitudinal association between weight misclassification at the 14‐year follow‐up with dieting up to the 30‐year follow‐up. Respondents who underestimated their body weight had approximately two times higher odds of dieting at each follow‐up (OR: 1.99; 95% CI: 1.19, 3.31) compared to those who correctly estimated their body weight. Respondents who overestimated their body weight had three times higher odds of dieting at each follow‐up (OR: 3.04; 95% CI: 1.90, 4.89) compared to their counterparts. After adjusting for potential confounders, these results remained significant. The association between misclassification and intermittent dieting behaviour groups appeared non‐significant.

**TABLE 3:** The association between weight misclassification at 14‐year with patterns of dieting to lose weight from 14‐year till 30‐year follow‐up (*N* = 1041) using data from Mater‐University of Queensland Study of Pregnancy.

Predictor	*N*	Not dieting	Dieting at 14 years, 21 years and 30 years F/U		Dieting at 21 years and 30 years F/U		Dieting at 21 years F/U		Dieting at 30 years F/U	
			Unadj. OR (95% CI)	Adjust. OR^a^ (95% CI)	Unadj. OR(95% CI)	Adjust. OR^a^ (95% CI)	Unadj. OR (95% CI)	Adjust. OR^a^ (95% CI)	Unadj. OR (95% CI)	Adjust. OR^a^ (95% CI)
Misclassification at 14 years F/U										
Correct estimation (reference)	632	1.00	1.00	1.00	1.00	1.00	1.00	1.00	1.00	1.00
Underestimation	196	1.00	1.99 (1.19, 3.31)*	1.90 (1.06, 3.39)*	0.98 (0.61, 1.58)	0.90 (0.51, 1.57)	1.36 (0.76, 2.43)	1.32 (0.69, 2.53)	1.09 (0.71, 1.69)	1.05 (0.66, 1.67)
Overestimation	213	1.00	3.04 (1.90, 4.89)*	2.20 (1.32, 3.68)*	1.23 (0.77, 1.95)	0.91 (0.55, 1.50)	1.37 (0.76, 2.49)	1.06 (0.57, 2.0)	1.32 (0.86, 2.02)	1.22 (0.78, 1.91)

^a^ Adjusted OR: Sex, race, maternal education, maternal BMI at baseline follow‐up; exercise, family eating together and pubertal development at 14 years of age.

* Results are statistically significant with *p* value < 0.05.

Additional analyses showed the independent association between weight misclassification and dieting at each follow‐up across body weight categories. In stratified analyses, findings differed by baseline BMI categories. After accounting for all covariates, weight underestimation among adolescents with normal weight was associated with lower odds of dieting at the 21‐year follow‐up in both unadjusted and adjusted models. Similarly, weight overestimation among normal weight adolescents remained significantly associated with dieting across all follow‐up periods. These findings are presented in Tables S2 and S3. By definition, overestimation was not defined among respondents with overweight or obesity.

## 4 Discussion

The results of this Australian longitudinal study suggest that body weight misclassification in adolescence is associated with dieting behaviours during adolescence and may influence dieting patterns from adolescence to adulthood. Adolescents who misclassify their body weight are at an increased risk of engaging in dieting during the adolescent period as well as in adulthood. This study also showed that weight status overestimation is associated with considerably higher odds of long‐term dieting behaviour than the underestimation of body weight. Underestimation of body weight can further increase the already higher risk of obesity in adolescents experiencing higher than healthy body weight [3, 14, 15]. Overestimation of body weight, on the other hand, can lead to adverse health outcomes due to inappropriate weight control behaviours. These findings indicate that misclassification of body weight in early adolescence may contribute to the initiation and continuation of dieting, including potentially harmful dieting behaviours. Therefore, health promotion programmes focusing on adolescents' education to promote more accurate body weight perception, foster positive body image, healthy food behaviours, critical awareness of sociocultural pressures and engagement in balanced and sustainable health behaviours may play a role in improving wellbeing and reducing both weight misclassification and potentially harmful dieting practices. Importantly, such programmes may also address the sociocultural pressures related to body size that contribute to psychological distress and engagement in unhealthy eating patterns.

This study points to factors that are associated with dieting which are, body weight misclassification as well as its components (i.e., BMI and self‐perception of body weight). Respondents who were overweight and obese were more likely to engage in dieting compared to those who were underweight. Similarly, regardless of respondents' body weight, those who perceived their body weight as overweight and obese were more likely to engage in dieting compared to those who perceived their body weight as underweight. Also, respondents who overestimated their body weight reported dieting more often at each follow‐up compared with those who correctly or underestimated their weight. These results showed that a substantial proportion of those engaging in dieting behaviours during the adolescent period continued to report dieting in adulthood, which aligns with previous studies that have reported persistence of dieting behaviours over time [24, 25].

Adolescents who overestimated their body weight had higher odds of dieting behaviour at the 14‐year follow‐up in both unadjusted and adjusted models. However, these associations were not statistically significant at the adult follow‐ups after adjusting for covariates. This pattern suggests that weight overestimation during adolescence may be associated with dieting at that stage, while the evidence for persistent associations into adulthood appears less consistent after accounting for potential confounders.

Although body weight overestimation has been considered a protective factor against weight gain as it is associated with weight control behaviours, some studies have reported associations with unhealthy weight control behaviours including the use of diet pills and laxatives [18, 36]. Body weight underestimation has been reported to be associated with lesser effort at weight control behaviours. It is known that obesity and being overweight are associated with weight control behaviours [37, 38], a finding reinforced in this study. In addition, it would be expected that dieting may be less common among those who underestimate their body weight. Considering this expectation, additional analysis showed that weight underestimation was associated with lower odds of dieting at the 21‐year follow‐up among normal weight respondents (in both unadjusted and adjusted models) and at the 14‐year and 21‐year follow‐ups among respondents with overweight and obesity; however, among the overweight and obesity group, these associations were no longer significant after adjusting for covariates.

Research on body weight misclassification and dieting suggests that both factors might be associated with future weight gain [19, 30, 39, 40]. Body weight concern and dieting may be established during adolescent period [41, 42, 43]. Body weight misclassification and weight‐related concerns are potentially influenced by sociocultural pressures, media exposure and internalised body weight ideals, which may distort perceived weight status and motivate unnecessary dieting [43, 44, 45]. This study suggests that weight overestimation among normal weight adolescents is associated with dieting practice across adolescence and adulthood. Thus, addressing weight overestimation and dieting as early as the adolescent period might contribute to promoting healthy food behaviours in adulthood. Effective strategies may include school‐based health promotion, media literacy programmes and interventions that promote accurate weight perception and positive body image, while discouraging weight‐based stigma and unhealthy weight‐control practices [44].

To our knowledge, this study is among the first to study the longitudinal association between body weight misclassification as a predictor and dieting from the adolescent to adult period of the life course as an outcome. This study has several advantages such as a large sample and longitudinal design, which obtains measurements from critical life stages. Also, this is a population‐based cohort study with results that are likely to be generalizable to other population‐based samples. However, use of BMI alone as the sole measure of obesity and missing values are the main limitations that could affect the study findings. BMI cannot be considered as a diagnostic test or measurement tool for obesity or fatness in this study. While missing values can alter the study results, previous MUSP research has found that missing data do not significantly impact research findings, even after applying inverse probability weightings and multiple imputation methods [23]. Also, MUSP research shows that those who remained in the study and those who abandoned follow‐up had similar baseline characteristics associations across the study period phases [22, 23, 46, 47]. Another limitation of this study is that we could not differentiate between healthy and unhealthy dieting practices. Further research is needed to compare the longitudinal impact of weight misclassification on healthy and unhealthy dieting behaviours. Finally, the dieting variable was divided into two response categories (dieting vs. not dieting) at each follow‐up because the questions and the response options were phrased differently at the 14‐year follow‐up compared with those at the 21‐year and 30‐year follow‐ups. Additionally, in this research, weight misclassification could only be assessed at the 14‐year follow‐up due to substantial missing data at later follow‐ups. Future studies examining longitudinal associations between weight misclassification and dieting behaviours should, where possible, include repeated assessments of weight misclassification across follow‐up periods as a covariate in their multivariable models.

## 5 Conclusion

Overall, misclassification of body weight seems to play an important role in promoting adolescence and adulthood dieting behaviour. Interventions that promote accurate body weight perception and support healthy dieting patterns may mitigate the risks associated with body weight misclassification. Our findings therefore should not be interpreted as endorsing dieting as inherently beneficial, but rather as highlighting how weight misclassification can trigger behaviours that carry potential health behaviour risks. Further research is needed to develop and evaluate health programmes for promoting positive body image, reducing body weight misclassification and its burden on dieting practices and eating disorders from adolescence to adulthood.

## Funding

This research received no specific grant from any commercial funding agency. The study has been funded by government awarded competitive research grants from the National Health and Medical Research Council (NHMRC) and the Australian Research council. All this project proposal and procedures involving this project study participants were approved by The University of Queensland ethical clearance [grant numbers B/88/Anth/94/NHMRC; B/660/SS; 1525A] and Mater Misericordiae Children's Hospital ethical clearance [grant numbers 506A; 1735A].

## Ethics Statement

Approvals to conduct each follow‐up were obtained either from the University of Queensland and/or Mater Hospital Human Research Ethics Committees, which are consistent with the Declaration of Helsinki statement.

## Conflicts of Interest

The authors declare no conflicts of interest.

## Supporting information


**TABLE S1:** List of covariates that have potential influence on dieting behaviours of adolescent.
**TABLE S2:** The association between weight misclassification at 14‐year follow‐up, among normal weight group [10] and dieting to lose weight at 14‐year, 21‐year and 30‐year follow‐ups (*N* = 773) using data from Mater‐University of Queensland Study of Pregnancy.
**TABLE S3:** The association between weight misclassification at 14‐year follow‐up, among overweight [10] and dieting to lose weight at 14‐year, 21‐year and 30‐year follow‐ups (*N* = 244) using data from Mater‐University of Queensland Study of Pregnancy.

## Data Availability

The raw data and analyses from this study are subject to access restrictions in accordance with the confidentiality requirements outlined in the ethics committee's approval statement. However, the data may be made available upon reasonable request and in accordance with the committee's conditions.
