# Supplementary material for: The Impact of Adolescent Body Weight Misclassification on Dieting Behaviour From Adolescence to Adulthood: A Longitudinal Study
Source: Health Promot J Austr. 2026 Apr 14;37(2):e70185. doi: 10.1002/hpja.70185 (PMC13078966; doi:10.1002/hpja.70185)
Supplement: Supplementary file 1 — TABLE S1: List of covariates that have potential influence on dieting behaviours of adolescent. TABLE S2: The association between weight misclassification at 14‐year follow‐up, among normal weight group [10] and dieting to lose weight at 14‐year, 21‐year and 30‐year follow‐ups (N = 773) using data from Mater‐University of Queensland Study of Pregnancy. TABLE S3: The association between weight misclassification at 14‐year follow‐up, among overweight [10] and dieting to lose weight at 14‐year, 21‐year and 30‐year follow‐ups (N = 244) using data from Mater‐University of Queensland Study of Pregnancy. [file HPJA-37-0-s001.docx]

| Maternal body weight ^1, 2^ |
| --- |
| Maternal education ^3^ |
| Racial origin ^4, 5^ |
| Gender ^6^ |
| Physical activity ^4, 7^ |
| Pubertal development ^8^ |
| Family eating behaviours ^9^ |

Table S1: List of covariates that has potential influence on dieting behaviours of adolescent

1. van den Berg PA, Keery H, Eisenberg M, Neumark-Sztainer D. Maternal and adolescent report of mothers' weight-related concerns and behaviors: longitudinal associations with adolescent body dissatisfaction and weight control practices. J Pediatr Psychol. 2010;35(10):1093-102.

2. Maximova K, McGrath JJ, Barnett T, O'Loughlin J, Paradis G, Lambert M. Do you see what I see? Weight status misperception and exposure to obesity among children and adolescents. Int J Obes (Lond). 2008;32(6):1008-15.

3. Niemeier BS, Hektner JM, Enger KB. Parent participation in weight-related health interventions for children and adolescents: A systematic review and meta-analysis. Preventive Medicine. 2012;55(1):3-13.

4. Chung AE, Perrin EM, Skinner AC. Accuracy of child and adolescent weight perceptions and their relationships to dieting and exercise behaviors: a NHANES study. Acad Pediatr. 2013;13(4):371-8.

5. Duncan DT, Wolin KY, Scharoun-Lee M, Ding EL, Warner ET, Bennett GG. Does perception equal reality? Weight misperception in relation to weight-related attitudes and behaviors among overweight and obese US adults. Int J Behav Nutr Phys Act. 2011;8:20.

6. Yan AF, Zhang G, Wang MQ, Stoesen CA, Harris BM. Weight perception and weight control practice in a multiethnic sample of US adolescents. South Med J. 2009;102(4):354-60.

7. Hill AJ. Does dieting make you fat? British Journal of Nutrition. 2004;92(S1):S15-S8.

8. McCabe MP, Ricciardelli LA. Sociocultural influences on body image and body changes among adolescent boys and girls. J Soc Psychol. 2003;143(1):5-26.

9. Neumark-Sztainer D, Bauer KW, Friend S, Hannan PJ, Story M, Berge JM. Family weight talk and dieting: how much do they matter for body dissatisfaction and disordered eating behaviors in adolescent girls? J Adolesc Health. 2010;47(3):270-6.

10. Cole TJ, Bellizzi MC, Flegal KM, Dietz WH. Establishing A Standard Definition For Child Overweight And Obesity Worldwide: International Survey. BMJ: British Medical Journal. 2000;320(7244):1240-3.

Table S2: The association between weight misclassification at 14-year follow-up, among normal weight group ^10^, and dieting to lose weight at 14-year, 21-year, and 30-year follow-ups (N=773) using data from Mater-University of Queensland Study of Pregnancy.

| **Predictor** | **N** | **Not dieting** | **Dieting at 14y F/U^†^** | | **Dieting at 21y F/U^†^** | | | **Dieting at 30y F/U^†^** | |
| --- | --- | --- | --- | --- | --- | --- | --- | --- | --- |
|  |  |  | **Dieting** | | **Dieting** | | | **Dieting** | |
|  |  |  | *Unadj. OR*  *(95% CI)* | *Adjust. OR*^‡^  *(95% CI)* | | *Unadj. OR*  *(95% CI)* | *Adjust. OR*^‡^  *(95% CI)* | *Unadj. OR*  *(95% CI)* | *Adjust. OR*^‡^  *(95% CI)* |
| **Misclassification at 14y F/U** |  |  |  |  | |  |  |  |  |
| Correct estimation | 500 | 1.00 | 1.00 | 1.00 | | 1.00 | 1.00 | 1.00 | 1.00 |
| Underestimation | 82 | 1.00 | 1.15 (0.47, 2.85) | 0.94 (0.34, 2.55) | | 0.52 (0.28, 0.95)* | 0.35 (0.17, 0.72)* | 0.61 (0.36, 1.01) | 0.64 (0.37, 1.11) |
| Overestimation | 191 | 1.00 | 8.85 (5.57, 14.05)* | 7.02 (4.25, 11.60)* | | 2.71 (1.92, 3.83)* | 1.69 (1.13, 2.54)* | 2.26 (1.61, 3.18)* | 1.93 (1.33, 2.79)* |

**^†^**F/U: follow-up

**^‡^***Adjusted OR*: Sex, race, maternal education, maternal BMI at baseline follow-up; exercise, family eating together and pubertal development at 14 years of age

*Results are statistically significant with P-value<0.05

1. Cole TJ, Bellizzi MC, Flegal KM, Dietz WH. Establishing A Standard Definition For Child Overweight And Obesity Worldwide: International Survey. BMJ: British Medical Journal. 2000;320(7244):1240-3 DOI: <https://doi.org/10.2307/25224434>.

Table S3: The association between weight misclassification at 14-year follow-up, among overweight ^10^, and dieting to lose weight at 14-year, 21-year, and 30-year follow-ups (N=244) using data from Mater-University of Queensland Study of Pregnancy.

| **Predictor** | **N** | **Not dieting** | **Dieting at 14y F/U^†^** | | **Dieting at 21y F/U^†^** | | **Dieting at 30y F/U^†^** | |
| --- | --- | --- | --- | --- | --- | --- | --- | --- |
|  |  |  | **Dieting** | | **Dieting** | | **Dieting** | |
|  |  |  | *Unadj. OR*  *(95% CI)* | *Adjust. OR*^‡^ *(95% CI)* | *Unadj. OR*  *(95% CI)* | *Adjust. OR***^‡^** *(95% CI)* | *Unadj. OR*  *(95% CI)* | *Adjust. OR***^‡^** *(95% CI)* |
| **Misclassification at 14y F/U** |  |  |  |  |  |  |  |  |
| Correct estimation | 119 | 1.00 | 1.00 | 1.00 | 1.00 | 1.00 | 1.00 | 1.00 |
| Underestimation | 125 | 1.00 | 0.52 (0.31, 0.86)* | 0.61 (0.33, 1.10) | 0.58 (0.34, 0.98)* | 0.69 (0.36, 1.30) | 0.67 (0.39, 1.17) | 0.64 (0.34, 1.20) |

**^†^**F/U: follow-up

**‡***Adjusted OR*: Sex, race, maternal education, maternal BMI at baseline follow-up; exercise, family eating together and pubertal development at 14 years of age

*Results are statistically significant with P-value<0.05

1. Cole TJ, Bellizzi MC, Flegal KM, Dietz WH. Establishing A Standard Definition For Child Overweight And Obesity Worldwide: International Survey. BMJ: British Medical Journal. 2000;320(7244):1240-3 DOI: <https://doi.org/10.2307/25224434>.
